# Bioefficacy of *Lantana camara* L. against Some Human Pathogens

**DOI:** 10.4103/0250-474X.58177

**Published:** 2009

**Authors:** B. Sharma, P. Kumar

**Affiliations:** Laboratory of Plant Tissue Culture and Secondary Metabolites, Department of Botany, University of Rajasthan, Bapu Nagar, Jaipur-302 004, India

**Keywords:** Alkaloid, flavonoid, MIC, MBC/MFC, Total activity

## Abstract

Antimicrobial efficacy of flavonoids (free and bound) and crude alkaloids of *Lantana camara* L. was determined by disc diffusion assay against three bacteria (*Escherichia coli, Proteus mirabilis,* and *Staphylococcus aureus*) and two fungi (*Candida albicans* and *Trichophyton mentagrophytes*). Minimum inhibitory concentration, minimum bactericidal/fungicidal concentration and total activity were also studied. Most susceptible microorganism in the present study was *C. albicans* followed by *P. mirabilis, S. aureus, E. coli,* and *T. mentagrophytes.* The range of minimum inhibitory concentration of tested extracts was 0.039-0.625 mg/ml while minimum bactericidal/fungicidal concentration ranged from 0.078-1.25 mg/ml. Six extracts out of eleven tested showed same values of minimum inhibitory concentration and minimum bactericidal/fungicidal concentration, while rest showed higher values of minimum bactericidal/fungicidal concentration. Highest total activity (120.51 ml/g) was observed for bound flavonoids of root against *Candida albicans* and *Staphylococcus aureus*. Results of the present investigation indicate that *Lantana camara* has good antimicrobial activity with low range of minimum inhibitory concentration hence can be exploited for future plant based antimicrobial drugs.

*Lantana camara* L. (Verbenaceae) is a perennial shrub, brought to India some 80 years ago from South America, which has become exotic and spread to different regions of the country. All the parts of this plant are traditionally used for several ailments. Leaves of the plant are antiseptic, antitumoural, and antimicrobial[1] whereas, roots are used in the treatment of malaria, rheumatism, and skin rashes[2]. Selected microorganisms are responsible for several ailments in human beings viz. *E. coli* and *P. mirabilis* are known to cause diarrhoea, urinary tract infections and sepsis, while major cause of nosocomial, suppurative infections and food poisoning is *S. aureus*. Similarly, candidiasis is caused by *C. albicans* and *T. mentagrophytes* is known to cause dermatophytosis.

Drug resistance developing in pathogenic microorganisms against commonly used antibiotics has necessitated a search for new antimicrobial compounds from biological sources (plants). In the present study efforts have been made to evaluate the antimicrobial potential of flavonoids and crude alkaloid extracts of *L. camara* against five human pathogens.

Different parts (flower, leaf, stem, and root) of *L. camara* were collected from Jaipur, (India) and was identified at the Department of Botany, University of Rajasthan and sample specimen No. RUBL 20354 was submitted in the herbarium of Botany Department, University of Rajasthan. Plant parts were separately shade dried and finely powdered using a blender.

Flavonoids were extracted from root, stem, leaf and flower of *L. camara* following the well established method of Subramanian and Nagarjan[3]. Hundred grams of each finely powdered sample was Soxhlet extracted with 80% hot methanol (500 ml) on a water bath for 24 h and filtered. Filtrate was re-extracted successively with petroleum ether, ethyl ether, and ethyl acetate. Petroleum ether fractions were discarded as being rich in fatty substances. Ether and ethyl acetate fractions containing free and bound Flavonoids respectively were dried *in vaccuo*.

Finely powdered sample (100 g) of plant parts (root, stem and leaf) were extracted with 10% acetic acid in ethanol (final volume 500 ml) for 4 h. Extracts were then concentrated to ¼ of the original volume and NH_4_OH was added drop wise. Precipitate collected after centrifugation, washed with NH_4_OH and dried *in vaccuo*[4].

Pathogenic microorganisms under study were two Gram negative bacteria *Escherichia coli* (MTCC 46), *Proteus mirabilis* (MTCC 1425), one Gram positive bacteria *Staphylococcus aureus* (MTCC 87), a yeast *Candida albicans* (MTCC 183), and a dermatophytic fungi *Trichophyton mentagrophytes* (MTCC 7687). Selected microorganisms were procured from MTCC, IMTECH, Chandigarh, India. Bacterial and fungal strains were grown and maintained on Muller-Hinton agar medium' and Sabouraud dextrose agar medium, respectively.

Disc Diffusion assay was carried out for determination of antimicrobial activity of the extracts[5]. Filter paper discs of extracts and standard drug (1 mg) were prepared and screened against selected pathogens. Each extract was assayed in triplicate. Antimicrobial efficacy of the extracts was evaluated by inhibition zone diameter and activity index. Microbroth dilution method[6] with slight modifications was followed for determination of minimum inhibitory concentration (MIC). Plant extracts were resuspended in acetone (which has no activity against selected pathogens) to make stock solutions of 5 mg/ml concentrations which were then two fold serially diluted in 96- well microtitre plates. Bacterial and fungal suspensions were used as negative control, while broth containing extracts were used as positive control. Experiments were carried out in duplicates and each time two sets were prepared, one was kept for incubation while other set was kept at 4° for comparing the turbidity in the wells of microtitre plate.

MIC values were taken as the lowest concentration of the extracts in the wells of microplate that showed no turbidity after incubation. The turbidity in the wells of microplate indicated visible growth of microorganisms. The minimum bactericidal/ fungicidal concentration (MBC/MFC) evaluated by subculturing 50 μl solution from each well showing no turbidity i.e. no growth of microorganisms. Least concentration of extract showing no visible growth on subculture was taken as MBC/MFC. Total activity is the volume at which test extract can be diluted with the ability to kill microorganisms. The total activity in ml/g was calculated by dividing the MIC value with the quantity extracted from 1 g of plant material[7].

Antimicrobial potency of flavonoids (free and bound) and crude alkaloids of *L. camara* was assessed by inhibition zone, activity index (Table 1), minimum inhibitory concentration and minimum bactericidal/fungicidal concentration (Table 2). Quantity of extracts per gram of plant material was also calculated (Table 3). In the present investigation total 11 extracts were tested, among which 9 extracts showed antimicrobial activity. Three extracts were found inactive against all the test pathogens at chosen concentration range. All selected five pathogens were inhibited by bound flavonoids of leaves. Most of the extracts showed bioactivity against more than two microorganisms tested. Bound flavonoids were found to be more potent than free flavonoids of the selected plant.

**TABLE 1:** DETERMINATION OF IZ AND AI VALUES OF EXTRACTS OF *LANTANA CAMARA* L.

Microorganisms→		*E. coli*		*S. aureus*		*P. mirabilis*		*C. albicans*		*T. mentagrophytes*	
						
Plant Part	Extract	IZ	AI	IZ	AI	IZ	AI	IZ	AI	IZ	AI
Root	A	-	-	-	-	-	-	-	-	-	-
Root	B	-	-	19.3±0.67	0.77±0.03	17.7±0.33	0.74±0.02	31.7±0.88	3.2±0.09	22.3±0.3	0.49±0.01
Root	C	-	-	7.7±0.33	0.31±0.01	-	-	7.66±0.33	0.77±0.033	-	-
Stem	A	7.7±0.67	0.38±0.033	-	-	13.3±0.67	0.56±0.027	9.0±0.58	0.93±0.03	-	-
Stem	B	-	-	-	-	-	-	-	-	-	-
Stem	C	9.0±0.58	0.45±0.03	-	-	8.3±0.88	0.35±0.04	-	-	-	-
Leaf	A	-	-	-	-	-	-	-	-	-	-
Leaf	B	9.3±0.33	0.47±0.02	15.3±0.67	0.613±0.03	14.0±0.58	0.58±0.02	9.3±0.33	0.93±0.03	8.3±0.03	0.19±0.01
Leaf	C	-	-	-	-	-	-	9.3±0.33	0.93±0.03	-	-
Flower	A	-	-	8.7±0.33	0.35±0.01	-	-	-	-	-	-
Flower	B	15.3±0.3	0.77±0.02	17.7±0.33	0.71±0.01	15.7±09	0.65±0.04	8.7±0.33	0.87±0.03	-	-

IZ= inhibition zone (mm; including 6 mm diameter of disc) -: no activity of extract, A is free flavonoids fraction, B is bound flavonoids fraction and C is crude alkaloid fraction; AI= activity Index (IZ produced by extract/IZ produced by standard). IZ of standard drug streptomycin against *E. coli* (20 mm), *S. aureus* (25 mm), *P. mirabilis* (24 mm), and IZ of terbinafine against *C. albicans* (10 mm) and *T. mentagrophytes* (45 mm), ±SEM

**TABLE 2:** DETERMINATION OF MIC AND MBC/MFC VALUES OF EXTRACTS OF *LANTANA CAMARA* L.

Microorganisms		*E. coli*		*S. aureus*		*P. mirabilis*		*C. albicans*		*T. mentagrophytes*	
					
Plant Part	Extract	MIC	MBC	MIC	MBC	MIC	MBC	MIC	MFC	MIC	MFC
Root	A	-	-	-	-	-	-	-	-	-	-
Root	B	-	-	0.039	0.156	0.078	0.156	0.039	0.078	0.078	0.078
Root	C	-	-	0.625	1.25	-	-	0.625	1.25	-	-
Stem	A	0.625	1.25	-	-	0.156	0.312	0.312	0.625	-	-
Stem	B	-	-	-	-	-	-	-	-	-	-
Stem	C	0.312	0.625	-	-	0.625	0.625	-	-	-	-
Leaf	A	-	-	-	-	-	-	-	-	-	-
Leaf	B	0.312	0.625	0.156	0.312	0.156	0.312	0.312	0.625	0.625	0.625
Leaf	C	-	-	-	-	-	-	0.312	0.625	-	-
Flower	A	-	-	0.625	1.25	-	-	-	-	-	-
Flower	B	0.156	0.156	0.078	0.156	0.078	0.078	0.625	0.625	-	-

A is free flavonoids fraction, B is bound flavonoids fraction and C is crude alkaloid fraction, MIC: Minimum Inhibitory Concentration (mg/ml), MBC/MFC: Minimum Bactericidal/ Fungicidal Concentration (mg/ml)

**TABLE 3:** TOTAL ACTIVITY OF THE EXTRACTS OF *LANTANA CAMARA* L.

Plant Part	Extract	Quantity of extract mg/g dried plant part	Total Activity (ml/g)				

			*E. coli*	*S. aureus*	*P. mirabilis*	*C. albicans*	*T. mentagrophytes*
Root	A	5.4	-	-	-	-	-
Root	B	4.7	-	120.51	60.25	120.51	60.25
Root	C	4.1	-	6.56	-.	6.56	-
Stem	A	6.2	9.92	-	39.74	19.87	-
Stem	B	1	-	-	-	-	-
Stem	C	0.75	2.40	-	1.2	-	-
Leaf	A	1.51	-	-	-	-	-
Leaf	B	1.2	3.84	7.69	7.69	3.84	1.92
Leaf	C	0.82	-	-	-	2.62	-
Flower	A	1.34	-	2.14	-	-	-
Flower	B	0.53	3.39	6.79	6.79	0.848	-

Total activity= weight of extract (mg/g plant material)/MIC (mg/ml) of extract; A is free flavonoids fraction, B is bound flavonoids fraction and C is crude alkaloid fraction.

Bound flavonoids of flower showed pronounced activity against *E. coli* (Inhibition Zone (IZ) 15.33±0.333 mm, Activity Index (AI) 0.766±0.017) and *P. mirabilis* (IZ 15.66±0.882 mm, AI 0.652±0.350) with low MIC values (0.156 mg/ml and 0.078 mg/ml, respectively). Bound flavonoids of root showed good activity against *C. albicans* (IZ 31.66±0.882 mm, AI 3.16±0.088) with MIC value of 0.078 mg/ml, whereas bound flavonoids of root and flower were found highly active against *S. aureus* (IZ 19.33±0.667 mm, AI 0.773±0.027 and IZ 17.66±0.333 mm, AI 0.706±0.013, respectively) with MIC values of 0.039 mg/ml and 0.078 mg/ml, respectively.

Most resistant microorganism observed under present investigation was *T. mentagrophytes* against which only two extracts exhibited bioactivity. Both extracts (bound flavonoids of root and leaves) were found fungicidal in nature by showing same values of MIC and MFC. Bactericidal/ fungicidal effect of bound flavonoids of flowers was recorded against *E. coli*, *P. mirabilis* and *C. albicans* and alkaloid of stem was found bactericidal against *P. mirabilis.* All active extracts were recorded bacteriostatic against *S. aureus.* TA (total activity) as a measure of potency was determined. Most potent extract under study was bound flavonoids of root, which showed high values of TA against *S. aureus*, *P. mirabilis, T. mentagrophytes* and *C. albicans* (Table 3).

Medicinal plants could be a good alternative source of costly antibiotics (against which microbes are developing resistance rapidly), as most of the medicinal plants are safe with little or no side effects, cost effective and have ability to affect a wide range of antibiotic resistant microorganisms. Present study is an effort towards this direction.

In the current investigation *L. camara* showed its antimicrobial potential against test pathogens, which are being involved in a number of human diseases. *Lantana camara* has previously been studied for antibacterial and antifungal activities, but still the literature available is meager. Antibacterial activity of *L. camara* has been demonstrated against phytopathogenic *Xanthomonas campestris*[8]. Crude extract of *L. camara* root has been found to be active against *Staphylococcus aureus, Bacillus cereus,* and *Cladosporium sphaerosperum*[9]. Petroleum ether extract and essential oil of the plant has been proved to be antimicrobial[10,11]. Antifungal activity of flowers[12] and cytotoxic activity of *L. camara* has been well documented[13].

Screening of the plant under investigation (*L. camara)* so far has not been worked out for flavonoids and alkaloids. Mostly the crude extracts have been screened, that too without MIC, MBC/MFC and TA determination. Such studies could only indicate their antimicrobial potential but are not helpful in establishing them as an antibiotic.

In the present study IZ, AI, MIC, MBC/MFC and TA have been evaluated for each extract. For most of the extracts MIC values recorded were very low, indicating strong bioefficacy of the plant. It is worth mentioned that the IZ of the extracts against *C. albicans* found to be more as compared to standard drug.

*Lantana camara* is considered to be a voracious and poisonous weed, as it spreads rapidly and chocks the native vegetation, spoils animal habitats, biodiversity, leads to shortage of fodder for herbivores, and exerts toxic effects when ingested by livestock[14–16], hence *L. camara* is regularly eradicated by the villagers. However, present study advocates its uses by pharmaceutical industries for preparing plant based antimicrobials drugs, rather than to throw the eradicated plants as garbage.
